# Genomic Epidemiology of African Swine Fever Virus Identified in Domestic Pig Farms in South Korea during 2019–2021

**DOI:** 10.1155/2024/9077791

**Published:** 2024-01-16

**Authors:** Oh-Kyu Kwon, Da-Won Kim, Jin-Hwa Heo, Ji-Yun Kim, Jin-Ju Nah, Ji-Da Choi, Dong-Wook Lee, Ki-Hyun Cho, Seong-Keun Hong, Yeon-Hee Kim, Hae-Eun Kang, Jung-Hoon Kwon, Yeun-Kyung Shin

**Affiliations:** ^1^Foreign Animal Disease Division, Animal and Plant Quarantine Agency, Gimcheon, Gyeongsangbuk-do, Republic of Korea; ^2^College of Veterinary Medicine, Kyungpook National University, Daegu, Republic of Korea

## Abstract

African swine fever (ASF), a contagious viral disease, poses a significant threat to the global swine industry. In South Korea, ASF outbreaks have occurred since 2019, highlighting the need for a comprehensive understanding of the epidemiology and genetic characterization of the circulating African swine fever viruses (ASFVs). We obtained 21 ASFV isolates from domestic pig farms and analyzed their whole-genome sequences using the Illumina MiniSeq. Phylogenetic analysis was conducted using the maximum likelihood and time-scaled approaches to determine the genetic relationships and evolutionary dynamics of the Korean ASFV isolates. Comparative analysis of the 21 ASFV genomes with the reference strain Georgia 2007/1 revealed that while Korean isolates shared 11 mutations, they also had 22 discrete mutations, including single nucleotide polymorphisms and insertion/deletion polymorphisms (Indels). Phylogenetic analysis indicated that all Korean isolates were within the Asian subgroup of ASFV genotype II but were further divided into at least three distinct subclusters. Spatiotemporal analysis indicated multiple introductions of ASFVs into South Korea, crossing the national border with North Korea. In addition, we observed putative self-recombination between MGF 505-9R and MGF 505-10R genes in the ASFV/Korea/Pig/Inje2/2021 strain. Our findings provide insights into the genetic variations and evolution of ASFVs on South Korean pig farms from 2019 to 2021, uncovering multiple introductions of ASFVs across the national border, and highlighting the need for enhanced disease control strategies.

## 1. Introduction

African swine fever (ASF) is a highly contagious viral disease that affects domestic pigs and wild boars and causes severe economic losses and trade disruptions in the swine industry worldwide. The disease is caused by the ASF virus (ASFV), a large double-stranded DNA virus belonging to the *Asfarviridae* family. ASFV exhibits a wide range of clinical symptoms and high mortality rates in domestic pigs, with acute forms characterized by high fever, depression, and hemorrhage [1]. ASFV genotyping is based on the analysis of specific genetic markers such as the major protein p72 encoded by the gene *B646L* and the central variable region (CVR) within *B602L* [2–6]. ASFV strains have been classified into 24 genotypes, with genotypes I and II found outside Africa [4, 7, 8].

Genotype I ASFV strains were first reported in Portugal in 1957 and have since been detected in several countries, including Spain, France, Italy, Brazil, the Dominican Republic, and Haiti in the 1960s and the 1970s [9, 10]. ASFs due to genotype I ASFV strains have since been eradicated in all countries, except Sardinia and Italy, where they were endemic [11], until China reported genotype I ASFV isolates in 2021 for the first time in Asia [12]. Genotype II strains were introduced into Georgia in 2007, which marked the first outbreak outside Africa, and spread throughout the Trans-Caucasian region and Europe in 2014 [13, 14]. In Asia, China reported its first ASF outbreak in a pig farm in 2018, followed by many countries, including Mongolia, Vietnam, Cambodia, North Korea, Laos, the Philippines, Myanmar, Timor-Leste, Indonesia, and India. Since 2007, most ASFVs reported in European and Asian countries are genotype II [15–27].

In South Korea, ASF was first detected in 2019 on a pig farm in Paju, Gyeonggi Province. By the end of 2021, 21 outbreaks were reported in Korea [28]. In a previous study that analyzed 12 gene markers using partial sequencing [28], all 21 ASFV strains isolated from affected pig farms were found to belong to p72 genotype II, serogroup 8 with intergenic region (IGR) 173R-I329L II and CVR 1 [28]. Notably, no tandem repeat sequence (TRS) insertions were detected in the IGR A179L-A137R and IGR MGF 505 9R/10R, and no variations were observed in the O174L, K145R, MGF 505-5R, CP204L, or Bt/Sj regions among the 21 Korean isolates. In addition, the analyzed genes of these isolates were identical to those of Georgia 2007/1, the Chinese strains Pig/HLJ/2018 and China/2018/AnhuiXCGQ, and the Vietnamese strain ASFV_NgeAn_2019. However, further analysis revealed that *X69R*, located in the J268L region of the 18^th^ isolate (Korea/Pig/Goseong/2021), had a single tyrosine (Y) insertion at position 209. A previous study [28] stated that this finding implies that there are slight variations in ASFVs circulating in South Korea from 2019 to 2021 and that the source of the virus responsible for the 18^th^ ASFV (Korea/Pig/Goseong/2021)-infected farm was different from those of the other 20 pig farms. However, the detailed epidemiology of ASFV outbreaks in South Korea has not yet been fully determined owing to the low resolution of genomic data.

Recently, next-generation sequencing (NGS) methods have been applied for whole-genome sequencing of viruses, including ASFV [29–32]. To further understand the epidemiology, transmission, and evolution of ASFV in South Korea, in this study, we conducted whole-genome sequencing of 21 strains isolated between 2019 and 2021 using NGS methods. We analyzed genetic polymorphisms among Korean ASFVs and conducted a spatiotemporal transmission analysis using a time-scaled phylogenetic tree.

## 2. Materials and Methods

### 2.1. Samples

A total of 21 outbreaks were identified in South Korea between 2019 and 2021 (14 in 2019, 2 in 2020, and 5 in 2021). Three outbreaks, each in 2019 and 2021, were detected through nationwide monitoring of pig farms, which was initiated following the initial ASF outbreak in September 2019. In addition, two positive pigs were detected during testing at the slaughterhouse in 2020, leading to the tracing and confirmation of its origin farm and a neighboring farm. The remaining 13 outbreaks were confirmed based on notifications from pig farmers reporting sick or deceased pigs. Samples (blood or spleen) from all 21 outbreaks were collected for further analysis. Real-time PCR confirmed the presence of ASFV (Table 1).

### 2.2. DNA Extraction and Real-Time PCR for ASFV Detection

Viral DNA was extracted from the samples using the Maxwell® RSC Total Nucleic Acid Kit and Maxwell RSC Whole Blood DNA Extraction Kit (Promega, Madison, WI, USA) according to the manufacturer's instructions. The extracted DNA was stored at −20°C until analysis. Real-time PCR targeting the *B646L* gene encoding p72 for the detection of ASFV genomic DNA was performed using Bio-Rad CFX-96 (Bio-Rad, Hercules, USA) as described in the World Health Organization for Animal Health (WOAH) Manual (World Organization for Animal Health) [33, 34].

### 2.3. Whole-Genome Sequencing

DNA sequencing libraries for Illumina MiniSeq (Illumina, San Diego, CA, USA) were prepared using the Illumina Nextra XT DNA Library Preparation Kit and the Nextra XT Index Kit v2 Set A (Illumina) according to the manufacturer's instructions. Target enrichment was performed using an Enzymatic Preparation Kit (Celemics, Seoul, Republic of Korea), and a library was prepared. The prepared genomic DNA library and capture probes were hybridized with the prepared genomic library and capture probes using the Celemics Target Enrichment Kit (Celemics). Capture probes were chemically synthesized to hybridize with the target region, and the captured regions were amplified by post-PCR to enrich the genomic DNA. Before sequencing, library quality was assessed using a BioAnalyzer 2100 (Agilent, Santa Clara, USA) with an Agilent Bioanalyzer DNA High Sensitivity kit (Agilent) and quantified using a dsDNA High Sensitivity Assay kit (Thermo Fisher Scientific) and Qubit 2.0 Fluorometer (Thermo Fisher Scientific). MiniSeq sequencing was conducted in 150 bp paired-end mode using the MiniSeq High Output Reagent kit (300-cycle) kit (Illumina) according to the manufacturer's instructions.

### 2.4. Data Analysis

Adapter sequences and low-quality sequencing reads with a quality score below 70 were trimmed using the BBDuk v38.84 program. Taxonomy classification using the KRAKEN2 program (https://ccb.jhu.edu/software/kraken2/) was used to determine the percentage of ASFV genome in the remaining reads. The trimmed sequencing reads were then assembled by performing reference mapping against ASFV Georgia 2007/1 (GenBank Accession number NC_044959) using Geneious Prime software (https://www.geneious.com/). To minimize erroneous mappings, a maximum of 10% mismatch was allowed during the reference mapping process. Consensus sequences were generated considering only sites with coverage depths >20. NGS and assembly data are summarized in Table 2. Owing to the limitations of our short-read sequencing system, we were unable to obtain sequences for the terminal inverted repeat regions at both ends of the genome. The whole-genome sequences are uploaded in GenBank (Table 1).

### 2.5. Variant Confirmation Using Sanger Sequencing

Conventional PCR was performed using region-specific primer pairs shown in Table S1 and TaKaRa PrimerSTAR HS DNA (TaKaRa, Shiga, Japan) to confirm the variant sequences in the 21^st^ ASFV (Korea/Pig/Inje2/2021) genome. The reaction was performed on a Bio-Rad CFX-96 instrument (Bio-Rad).

### 2.6. Phylogenetic Analysis

Whole-genome sequences of genotype II ASFVs available in GenBank (https://www.ncbi.nlm.nih.gov/genbank/; data accessed on 01-Feb-2023) were downloaded. Viruses exhibiting an unusually high number of mutations exceeding the typical rate of viral mutations were excluded because of the suspicion of sequencing errors. For efficient computation and visualization, we chose representative sequences from those redundantly reported in the same region and during similar times. In addition, we reduced the number of European ASFV sequences for clearer visualization, based on prior studies [29, 32], because these European viruses are phylogenetically distinct from the Korean isolates. A total of 64 reference genomes, including the Georgia 2007/1 strain, were selected for phylogenetic analysis (Table S2). The 21 ASFV genomes analyzed in this study, along with the reference genomes, were aligned using the Multiple Alignment using Fast Fourier Transform method and manually trimmed to equal lengths with Georgia 2007/1, with approximately 187,420 sites including gaps. G/C homopolymers and inverted terminal repeats, prone to sequencing errors, were excluded.

A maximum-likelihood phylogenetic tree was constructed using RaxML v8.2.7, employing the general time reversible (GTR) nucleotide substitution model. Bootstrap analysis with 500 replicates was used to assess the statistical support of the phylogenetic tree. Georgia 2007/1 was used as the root of the phylogenetic tree.

In addition, a time-scaled phylogenetic tree was constructed using the BEAST v1.10.4 program [35]. An uncorrelated relaxed clock model with gamma-distributed rate (GTR + *γ*) nucleotide substitution was used. Four Markov chain Monte Carlo runs, each comprising 150 million steps, were run in parallel. The parameters and trees were sampled every 10,000 steps, resulting in 40,000 parameter states and posterior trees. TRACER v1.5 was used to analyze the parameters, with 10% of each result discarded as burn-in [36]. All parameters had an effective sample size of greater than 200. A time-scaled maximum clade credibility tree was generated using TreeAnnotator v1.10.4 (https://beast.community/treeannotator) in BEAST and visualized using FigTree v1.4.3 (http://tree.bio.ed.ac.uk/software/figtree/). The ASFV/Korea/Pig/Inje2/2021 strain was excluded from the time-scaled phylogenetic analysis because of suspected putative recombination. Reference sequences that deviated from the normal mutation rate and considered as errors were excluded.

## 3. Results

### 3.1. Whole-Genome Comparative Analysis

Comparative analysis of the 21 ASFV whole-genome sequences with the Georgia 2007/1 reference strain sequence revealed 33 mutations, including single nucleotide polymorphisms (SNPs) and insertion/deletion polymorphisms (Indels), present in the 21 Korean isolates. The Inje2/2021 strain had multiple additional mutations in the MGF 500-9R gene. Of the SNPs identified, 17 were nonsynonymous, five were synonymous, and the remaining 11 were detected in IGRs that do not code for any protein. All Korean isolates shared 11 mutations, of which six were nonsynonymous: T26425C (N329S) in MGF 360-10L, A44576G (K323E) in MGF 505-9R, T134514C (N414S) in NP419L, T170862A (I195F) in I267L, a truncation mutation (stop codon) in MGF 110-1L at position C7059T (W197 ^*∗*^), and a frameshift deletion causing protein truncation at position 12578 in the ASFV G ACD 00190 gene. In addition, a GAATATATAG insertion was found in the IGR between the I73R and I329L genes in all Korean isolates, indicating that all Korean isolates belonged to the IGR II genotype, based on the TRS classification of the IGR between I73R and I329L. The GAATATATAG insertion was confirmed by Sanger sequencing in previous study [28].

**Figure 1:**
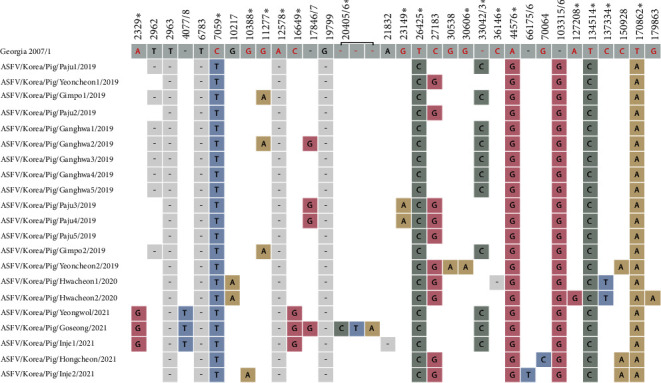
Mutations in whole-genome sequences of African swine fever viruses isolated from South Korea compared with the reference strain Georgia 2007/1. The nucleotide numbers are marked based on reference sequence (Georgia 2007/1). The location of the gaps in the reference sequence generated by nucleotide insertions of South Korea isolates is marked with a slash (/) between two nucleotide numbers. The sequences in open reading frames (ORFs) are highlighted in red in the reference sequence, whereas those located in intergenic regions (IGRs) are represented in black. Nonsynonymous mutations are marked with filled stars. Three nucleotide insertions between 20405 and 20406 are shown in the ASFV/Korea/Pig/Goseong/2021 strain. Mutations clustered in MGF 505-9R assumed to be the result of self-recombination in ASFV/Korea/Pig/Inje2/2021 and the GAATATATAG insertion at I73R-I329L IGR in all Korean isolates are not included in this figure.

A total of 22 genetic polymorphisms, including SNPs and Indels, were detected among Korean ASFVs; the nonsynonymous mutations are as follows: A2329G (L106P) mutation in MGF 360-1 La gene, G10388A (P114S) in MGF 110-7L, G11277A (A17V) in 285 L, C16649G (V243L) in MGF 460-4L, G23149A (A251V) in MGF 300-4L, G30606A (L268F) in MGF 300-12L, truncation (358 to 288aa) due to C insertion at 33042/3 in MGF 3605-14L, frameshift mutation due to C deletion at 36146 in MGF 505-3R, and C137334T (L506F) mutation in NP868R. The Goesong/2021 strain exhibited a frameshift mutation (70-71 aa) in the X69R gene owing to a CTA insertion at position 20405/20406. Two ASFVs detected in wild boars in Korea in 2019 and 2020 were included in the mutational investigation. The YC1/2019 strain (accession number, ON075797) detected in wild boars in the region near the demilitarized zone (DMZ), the national border between South and North Korea, in 2019, showed the same mutations as three ASFVs: Yeoncheon1/2019, Paju2/2019, and Paju5/2019 [37]. The HC224/2020 strain (accession number, OP628183) had identical mutations as YC1/2019 but had one more mutation, a 17846/7 G insertion in the noncoding region. Further details on the mutations identified in Korean ASFVs are shown in Figure 1 and Table S3.

**Figure 2:**
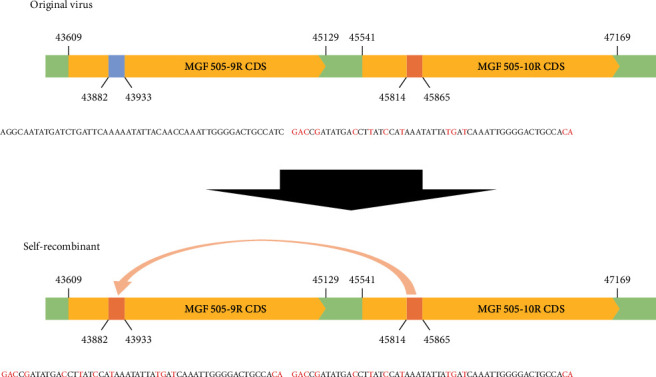
Diagram illustrating self-recombination in the ASFV/Korea/Pig/Inje2/2021 strain. Thirteen mutations were identified within the 52 bp segment of the MGF 505-9R gene and identical sequences were found in the MGF 505-10R gene. The original sequences (above) were identified in all other Korean isolates. The results indicated possible self-recombination between MGF 505-9R and MGF 505-10R in the ASFV/Korea/Pig/Inje2/2021 strain (below).

Notably, the Inje2/2021 strain exhibited numerous mutations concentrated in MGF 505-9R (13 mutations at nucleotide positions 43,882–43,934). Identical sequences were found at positions 45,814–45,866 in MGF 505-10R. The original sequences of MGF 505-9R (43,882–43,934) shared 75% identity with the MGF 505-10R gene, and nearby sequences also showed high homology (80.9%, nucleotide positions 43,874–43,941). These findings suggest that self-recombination may have occurred between the MGF 505-9R and MGF 505-10R genes in the ASFV/Korea/Pig/Inje2/2021 strain (Figure 2).

### 3.2. Phylogenetic Analysis

**Figure 3:**
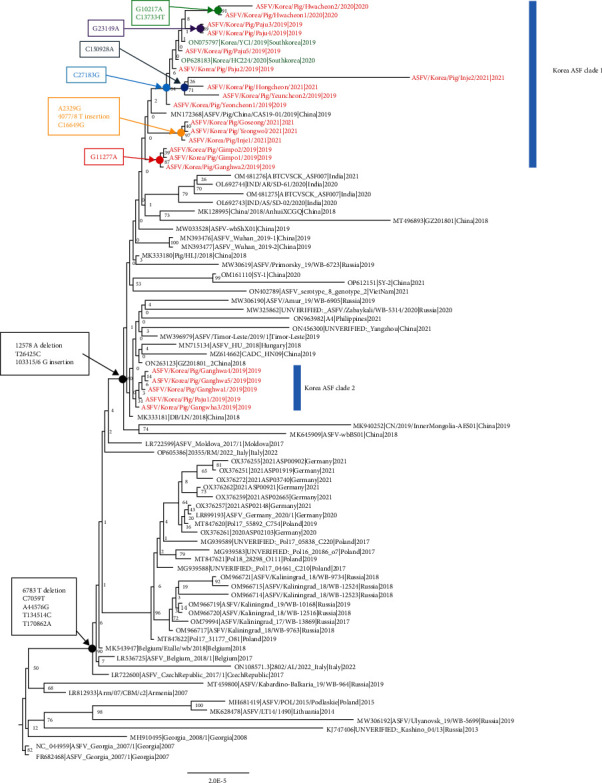
Maximum-likelihood phylogenetic tree of genotype II African swine fever viruses. Taxa with red font are the ASFV isolates from South Korean domestic pigs and taxa with green font are the ASFV isolates from South Korean wild boars. Phylogenetic trees were constructed using the maximum-likelihood method in RAxML with 500 bootstrap replicates. Bootstrap values are shown in each node. Each node sharing identical nucleotide mutations, insertion, or deletion among Korean isolates is annotated.

A phylogenetic tree was constructed to analyze the genomic epidemiology of ASFVs in South Korea. The maximum-likelihood phylogenetic tree revealed that genotype II ASFVs formed two distinct subgroups: Asian and European. All the Korean isolates clustered within the Asian subgroup (Figure 3). Although at least two distinct clusters specific to Korea were identified in the maximum-likelihood phylogenetic tree, most nodes in the tree did not receive strong bootstrap support (<70). Therefore, further investigation is required to explore the detailed genetic epidemiology of this maximum-likelihood phylogenetic tree.

**Figure 4:**
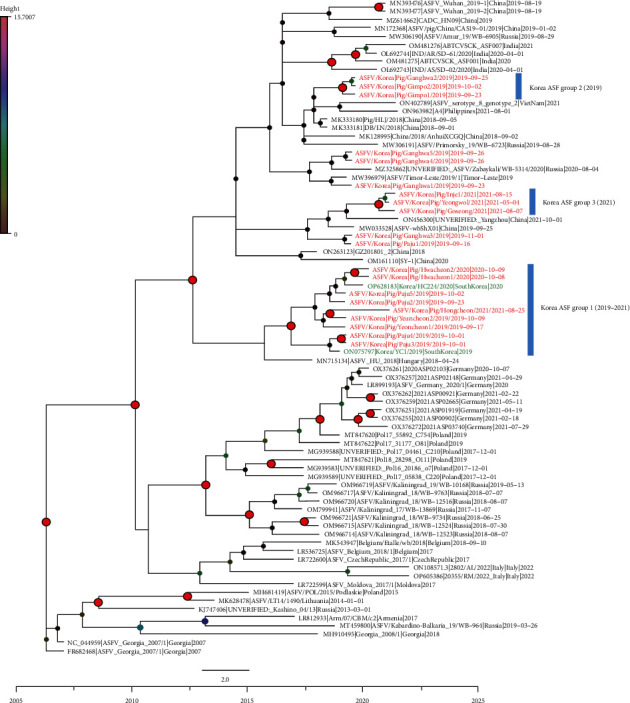
Time-scaled maximum clade credibility tree of the genotype II African swine fever viruses. Taxa with red font are the ASFV isolates from South Korean domestic pigs and taxa with green font are the ASFV isolates from South Korean wild boars. The size and the color of the node (circle) indicate the posterior, which means the probability associated with forming one clade. Korean ASF was divided into three groups according to the posterior probability.

**Figure 5:**
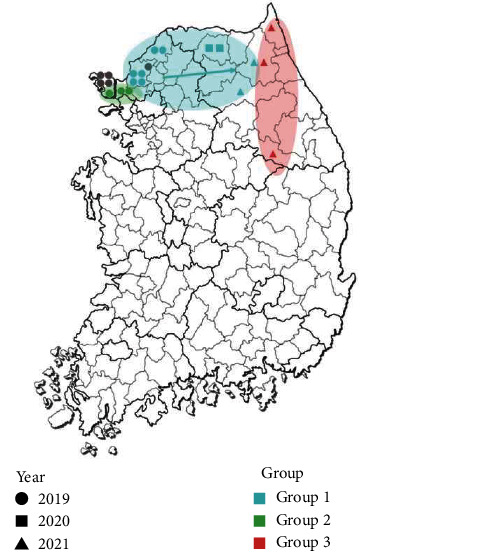
Distribution of ASFVs isolated in South Korea during 2019–2021 based on phylogenetic analysis. The shapes represent the year of isolation and the colors represent the viral groups in South Korea.

In addition, a time-scaled Bayesian phylogenetic tree was generated to gain further insights into the detailed genetic epidemiology of ASFVs in South Korea. The time-scaled phylogenetic tree revealed that the Korean ASFVs were divided into at least three subgroups, with each subgroup sharing a common node supported by a high posterior probability (>0.9) (Figure 4). Notably, each ASFV subgroup exhibited a geographical pattern (Figures 4 and 5). Viruses isolated from north Gyeonggi-do (Yeoncheon, Paju) in 2019 and west Gangwon-do (Hwacheon, Hongcheon) during 2020–2021 formed a cluster in the phylogenetic tree, designated as Korean subgroup I. This cluster also included two ASFV whole-genome sequences (YC1/2019 and HC224/2020) detected in wild boars in 2019 (Yeoncheon) and 2020 (Hwacheon). The virus isolated from west Gyeonggi-do (Gimpo, Ganghwa) was clustered in Korean subgroup II. Furthermore, viruses isolated from Gangwon-do (Yeongwol, Goseong, and Inje) formed a distinct cluster, designated as Korean subgroup III. These findings suggest that at least three distinct viruses were introduced into South Korea through west and north Gyeonggi-do and east Gangwon-do. Five other isolates detected in Gyeonggi-do (Ganghwa, Paju) did not cluster with the other Korean isolates with a high posterior probability. These phylogenetic outliers indicate the possibility of multiple introductions of ASFVs in South Korea.

## 4. Discussion

In this study, we conducted a comprehensive analysis of the whole-genome sequences of 21 ASFVs isolated in South Korea between 2019 and 2021. Through our analysis, we identified 33 mutations in the Korean isolates compared with the reference strain Georgia 2007/1. Of these, 17 nonsynonymous mutations, four substitutions (T26425C, A44576G, T134514C, and T170862A), one truncation (C7059T), and one frameshift mutation (A deletion at 12578) were consistent in all 21 Korean ASFVs. These mutations were also detected in ASFVs isolated from various Asian and European countries between 2007 and 2021 [29]. The A44576G (K323E) substitution in MGF-505-9R was documented in an ASFV isolate from Armenia as early as 2007. Similarly, truncation by C7059T and three substitutions (A44576G, T134514C, and T170862A) were present in ASFVs detected in several countries between 2017 and 2020. These results indicated that the mutations shared by the Korean isolates occurred before the virus was introduced into South Korea.

SNPs and Indels found in Korean isolates have also been detected in isolates from other countries. Frameshift mutations at 12578 and substitutions at T26425C have been detected in multiple countries and years. The frameshift mutation at position 12578 was more prevalent and was found in ASFVs from Lithuania, Poland, China, Vietnam, Russia, and Germany between 2014 and 2021. Substitution at T26425C was observed in ASFVs from China, Timor-Leste, Vietnam, Poland, and Armenia in 2018 and 2019. These findings suggest the possibility of multiple viral introductions into Korea, indicating that viruses were brought into the country at multiple instances, rather than mutations arising during local transmission. Furthermore, we identified a unique C insertion at position 33042/3 of the MGF 360-14L coding sequence. This insertion was observed in multiple Korean ASFVs from 2019 to 2021 as well as in ASFVs from Timor-Leste and Vietnam in 2019. The presence of this insertion in the Korean and international ASFVs suggests its potential significance as a relatively recent mutation. Proteins p30 (CP204L gene), p54 (E183L gene), and p72 (B646L gene) are suspected to be major epitopes for antibody-mediated protection. However, Korean isolates have not shown any mutations in these proteins in this study. Further studies are required to determine whether these mutations, particularly protein truncations, contribute to changes in the biological characteristics of the virus.

Our study also provides further support for the existence of distinct subgroups of ASFVs in Koreans, indicating multiple introductions of the virus over time. Through time-scale phylogenetic analysis, we found that groups 1 and 2 were initially isolated in Yeoncheon and Gimpo, respectively, and outlier groups that did not cluster with other Korean isolates were identified in Ganghwa and Paju. These findings suggest the continuous introduction of distinct viruses in areas near the DMZ. In group 3, the first outbreak was observed in Yeongwol, Gangwon-do, in 2021, a region geographically distant from the previously affected areas. Our phylogenetic analysis suggests the possibility of group 3 viruses being introduced through the DMZ near the east coast. However, it is important to acknowledge the limitations of our epidemiological investigation, particularly the absence of related reference sequences, including those from North Korea and wild boars. This leaves open the possibility that these clusters result from ASFV genetic mutations in wild boars, despite the virus's low mutation rate. Hence, global collaboration for the whole-genome sequencing of ASFVs from domestic pigs and wild boars is necessary to enhance our understanding of the epidemiology and transmission dynamics of the virus.

Recombination is a potential source of viral evolution, including changes in host range, virulence or pathogenesis, tissue tropism, resistance to antivirals, and the emergence of new viral diseases [38]. Previous studies have reported recombination events in ASFV, including homologous recombination, leading to genomic Indels that contribute to the genetic diversity of the virus [39], and recombination among different genotypes facilitated by the presence of recombination hotspots, resulting in the generation of diverse genetic strains [40]. In our study, we identified the possibility of self-recombination in the MGF 505 gene of the ASFV/Korea/Pig/Inje2/2021 strain. We detected a concentrated mutation in 13 SNPs within 52 bp of the MGF 505-9R genes, which shares an identical sequence with MGF 505-10R. Recombination could occur due to template switching by the polymerase among DNA or RNA strands that have high sequence identity [38]. Although MGF 505-9R and MGF 505-10R are paralogous proteins, their original sequences showed a total identity of approximately 61.4%. However, the MGF 505-9R showed 80.9% identity with that of MGF 505-10R in the region of putative recombination occurred. However, the precise mechanism of recombination between different gene locations in viruses not fully determined yet. Although Inje2/2021 exhibited potential self-recombination in MGF 505-9R, there were no discernible differences in virulence in pigs compared with the first Korean ASFV Paju1/2019 [28].

It is worth noting that although recombination events between different ASFV isolates have been reported, self-recombination of ASFV has not been previously documented [40, 41]. MGF 505/530 genes are believed to play important roles in virus tropism, virulence, and suppression of the interferon response, along with MGF 360 gene although the precise function of the encoded proteins is not fully understood [42]. Further studies are needed to elucidate the mechanisms underlying this recombination event and its implications for changes in biological characteristics, particularly the roles of proteins within individual viruses.

Despite the limitations of our study, primarily the lack of related reference sequences, genomic epidemiology using whole-genome sequences of ASFV provides valuable information on viral epidemiology. Therefore, continuous molecular epidemiological studies based on whole-genome sequences of ASFV are crucial for monitoring the origin of outbreaks and strengthening surveillance efforts. In this study, we present evidence for multiple possible introductions of ASFV through the DMZ in South Korea. These findings underscore the persistent challenge of repeated introduction of ASFVs into South Korea despite previous strain elimination of the virus through quarantine strategies. Therefore, intensive disease control measures are needed in regions such as Ganghwa-gun and Paju-si, where wild animals are likely to cross the DMZ, to prevent the introduction of new viruses.

In conclusion, our study provided valuable insights into the genetic diversity, mutations, and subgroups of ASFVs in South Korea. The identified mutations and subgroups suggest multiple introductions of ASFV strains into the country over time. These findings emphasize the need for intensified disease control measures, particularly in regions near the DMZ, to prevent the introduction of new viruses.

## Figures and Tables

**Table 1:** List of African swine fever viruses in samples analyzed in this study.

Sequence of outbreak	Strain	Collection date	Sample type	Location	Classification
1	ASFV/Korea/Pig/Paju1/2019	16/09/2019	Spleen	Paju	Farmer's notification
2	ASFV/Korea/Pig/Yeoncheon1/2019	17/09/2019	Spleen	Yeoncheon	Farmer's notification
3	ASFV/Korea/Pig/Gimpo1/2019	23/09/2019	Blood	Gimpo	Farmer's notification
4	ASFV/Korea/Pig/Paju2/2019	23/09/2019	Spleen	Paju	Farmer's notification
5	ASFV/Korea/Pig/Ganghwa1/2019	23/09/2019	Blood	Ganghwa	Active surveillance
6	ASFV/Korea/Pig/Ganghwa2/2019	25/09/2019	Blood	Ganghwa	Farmer's notification
7	ASFV/Korea/Pig/Ganghwa3/2019	25/09/2019	Blood	Ganghwa	Active surveillance
8	ASFV/Korea/Pig/Ganghwa4/2019	26/09/2019	Blood	Ganghwa	Farmer's notification
9	ASFV/Korea/Pig/Ganghwa5/2019	26/09/2019	Spleen	Ganghwa	Farmer's notification
10	ASFV/Korea/Pig/Paju3/2019	01/10/2019	Spleen	Paju	Farmer's notification
11	ASFV/Korea/Pig/Paju4/2019	01/10/2019	Blood	Paju	Active surveillance
12	ASFV/Korea/Pig/Paju5/2019	02/10/2019	Spleen	Paju	Farmer's notification
13	ASFV/Korea/Pig/Gimpo2/2019	02/10/2019	Spleen	Gimpo	Farmer's notification
14	ASFV/Korea/Pig/Yeuncheon2/2019	09/10/2019	Blood	Yeoncheon	Farmer's notification
15	ASFV/Korea/Pig/Hwacheon1/2020	08/10/2020	Spleen	Hwacheon	Active surveillance
16	ASFV/Korea/Pig/ Hwacheon2/2020	09/10/2020	Blood	Hwacheon	Active surveillance
17	ASFV/Korea/Pig/Yeongwol/2021	04/05/2021	Spleen	Yeongwol	Farmer's notification
18	ASFV/Korea/Pig/Goseong/2021	07/08/2021	Spleen	Goseong	Farmer's notification
19	ASFV/Korea/Pig/Inje1/2021	15/08/2021	Blood	Inje	Active surveillance
20	ASFV/Korea/Pig/Hongcheon/2021	25/08/2021	Blood	Hongcheon	Active surveillance
21	ASFV/Korea/Pig/Inje2/2021	05/10/2021	Spleen	Inje	Active surveillance

**Table 2:** Next-generation sequencing results of ASFV isolates analyzed in this study.

Strain	Total number of reads	Percent of reads mapped to ASFV genome^A,D^	Mean depth of coverage^B^	Genome coverage^C^ (%)	Recovered genome size	GenBank acc. number
ASFV/Korea/Pig/Paju1/2019	926,922	99.7	73	100	187,848	MT748042.2
ASFV/Korea/Pig/Yeoncheon1/2019	4,801,388	99.8	72.2	100	187,848	MW049116.2
ASFV/Korea/Pig/Gimpo1/2019	3,398,636	99.8	73	100	187,848	OR031244
ASFV/Korea/Pig/Paju2/2019	2,689,770	99.8	73.7	100	187,848	OR145817
ASFV/Korea/Pig/Ganghwa1/2019	2,426,070	99.8	73.8	100	187,848	OR145818
ASFV/Korea/Pig/Ganghwa2/2019	2,971,264	99.9	74.8	100	187,849	OR145819
ASFV/Korea/Pig/Ganghwa3/2019	1,467,574	99.8	74.2	100	187,848	OR145820
ASFV/Korea/Pig/Ganghwa4/2019	597,060	99.8	72.7	100	187,848	OR145821
ASFV/Korea/Pig/Ganghwa5/2019	1,817,732	99.8	60.8	100	187,848	OR145822
ASFV/Korea/Pig/Paju3/2019	3,416,744	99.8	73.3	100	187,849	OR145823
ASFV/Korea/Pig/Paju4/2019	2,400,358	99.7	69.1	100	187,849	OR145824
ASFV/Korea/Pig/Paju5/2019	3,785,414	99.7	77.5	99.9	187,848	OR145825
ASFV/Korea/Pig/Gimpo2/2019	2,521,942	99.4	75.4	100	187,848	OR145826
ASFV/Korea/Pig/Yeuncheon2/2019	3,556,236	99.6	79.9	100	187,848	OR145827
ASFV/Korea/Pig/Hwacheon1/2020	1,927,628	99.9	61.9	100	187,847	OR145828
ASFV/Korea/Pig/Hwacheon2/2020	1,256,522	62.2	48	99.2	187,848	OR145829
ASFV/Korea/Pig/Yeongwol/2021	5,396,554	96.7	66.4	100	187,850	OR145830
ASFV/Korea/Pig/Goseong/2021	5,228,860	34.8	72.2	99.7	187,854	OR145831
ASFV/Korea/Pig/Inje1/2021	8,490,160	14.9	47.7	99.2	187,849	OR145832
ASFV/Korea/Pig/Hongcheon/2021	6,711,708	26.2	77.9	100	187,848	OR145833
ASFV/Korea/Pig/Inje2/2021	8,774,068	50.7	71.6	99.9	187,849	OR145834

The number of total reads and percent of reads mapped to ASFV genome. ^A^Taxonomic classification of sequencing reads wereas assigned using KRAKEN2 program to calculate the percentage of ASFV genomes to the total reads. ^B^The mean number of reads per each nucleotide site. ^C^The percentage of the sequenced genome compared to with the whole-genome sequence. ^D^The four viruses detected in 2021 (Goseong/2021, Inje1/2021, Hongcheon/2021, and Inje2/2021) are targeted for enrichment in NGS.

## Data Availability

The whole-genome sequences used in this study were deposited in GenBank, and the accession numbers are provided in Table 1. Sequence alignments and results from phylogenetic analyses are available from the corresponding authors on reasonable request.

## Supplementary Materials

Supplementary Material Table S1: primers for Sanger sequencing of partial MGF-505-9R. Table S2: list of reference African swine fever virus sequences used in phylogenetic analysis. Table S3: summary of genetic variations detected between 21 Korean African swine fever viruses sequences and the reference strain, Georgia 2007/1 (NC_044959).

## References

[1] WOAH (2022). African swine fever (infection with African swine fever virus).

[2] Bastos A. D. S., Penrith M.-L., Crucière C. (2003). Genotyping field strains of African swine fever virus by partial p72 gene characterisation. *Archives of Virology*.

[3] Gallardo C., Fernandez-Pinero J., Pelayo V. (2014). Genetic variation among African swine fever genotype II viruses, eastern and central Europe. *Emerging Infectious Diseases*.

[4] Gallardo C., Reis A. L., Kalema-Zikusoka G. (2009). Recombinant antigen targets for serodiagnosis of African swine fever. *Clinical and Vaccine Immunology*.

[5] Ge S., Li J., Fan X. (2018). Molecular characterization of African swine fever virus, China, 2018. *Emerging Infectious Diseases*.

[6] Quembo C. J., Jori F., Vosloo W., Heath L. (2018). Genetic characterization of African swine fever virus isolates from soft ticks at the wildlife/domestic interface in Mozambique and identification of a novel genotype. *Transboundary and Emerging Diseases*.

[7] Boshoff C. I., Bastos A. D. S., Gerber L. J., Vosloo W. (2007). Genetic characterisation of African swine fever viruses from outbreaks in southern Africa (1973–1999). *Veterinary Microbiology*.

[8] Penrith M.-L., Vosloo W., Jori F., Bastos A. D. S. (2013). African swine fever virus eradication in Africa. *Virus Research*.

[9] Portugal R., Coelho J., Hoper D. (2015). Related strains of African swine fever virus with different virulence: genome comparison and analysis. *Journal of General Virology*.

[10] Sánchez-Vizcaíno J. M., Mur L., Martínez-López B. (2012). African swine fever: an epidemiological update. *Transboundary and Emerging Diseases*.

[11] Mur L., Martinez-Lopez B., Costard S. (2014). Modular framework to assess the risk of African swine fever virus entry into the European Union. *BMC Veterinary Research*.

[12] Sun E., Huang L., Zhang X. (2021). Genotype I African swine fever viruses emerged in domestic pigs in China and caused chronic infection. *Emerging Microbes & Infections*.

[13] Dixon L. K., Sun H., Roberts H. (2019). African swine fever. *Antiviral Research*.

[14] Rowlands R. J., Michaud V., Heath L. (2008). African swine fever virus isolate, Georgia, 2007. *Emerging Infectious Diseases*.

[15] Ankhanbaatar U., Sainnokhoi T., Khanui B. (2021). African swine fever virus genotype II in Mongolia, 2019. *Transboundary and Emerging Diseases*.

[16] Cadenas-Fernández E. D., Ito S., Aguilar-Vega C., Sánchez-Vizcaíno J. M., Bosch J. (2022). The role of the wild boar spreading African swine fever virus in Asia: another underestimated problem. *Frontiers in Veterinary Science*.

[17] Cooper T. L., Smith D., Gonzales M. J. C. (2022). Beyond numbers: determining the socioeconomic and livelihood impacts of African swine fever and its control in the Philippines. *Frontiers in Veterinary Science*.

[18] Denstedt E., Porco A., Hwang J. (2021). Detection of African swine fever virus in free-ranging wild boar in Southeast Asia. *Transboundary and Emerging Diseases*.

[19] Dharmayanti N. L. P. I., Sendow I., Ratnawati A. (2021). African swine fever in North Sumatra and West Java provinces in 2019 and 2020, Indonesia. *Transboundary and Emerging Diseases*.

[20] Khoo C. K., Norlina D., Roshaslinda D. (2021). African swine fever in backyard pigs of Sabah state, East Malaysia, 2021. *Tropical Biomedicine*.

[21] Kim S.-H., Kim J., Son K. (2020). Wild boar harbouring African swine fever virus in the demilitarized zone in South Korea, 2019. *Emerging Microbes & Infections*.

[22] Mai N. T. A., Vu X. D., Nguyen T. T. H. (2021). Molecular profile of African swine fever virus (ASFV) circulating in Vietnam during 2019–2020 outbreaks. *Archives of Virology*.

[23] Matsumoto N., Siengsanan-Lamont J., Halasa T. (2021). The impact of African swine fever virus on smallholder village pig production: an outbreak investigation in Lao PDR. *Transboundary and Emerging Diseases*.

[24] Mighell E., Ward M. P. (2021). African swine fever spread across Asia, 2018–2019. *Transboundary and Emerging Diseases*.

[25] Mileto P., da Conceição F., Stevens V. (2021). Complete genome sequence of African swine fever virus isolated from a domestic pig in timor-leste, 2019. *Microbiology Resource Announcements*.

[26] Rajkhowa T. K., Kiran J., Hauhnar L., Zodinpui D., Paul A., Sagolsem S. (2022). Molecular detection and characterization of African swine fever virus from field outbreaks in domestic pigs, Mizoram, India. *Transboundary and Emerging Diseases*.

[27] Tran H. T. T., Truong A. D., Dang A. K. (2021). Genetic characterization of African swine fever viruses circulating in North Central region of Vietnam. *Transboundary and Emerging Diseases*.

[28] Cho K.-H., Kim D.-Y., Jang M.-K. (2022). Genetic characterization of African swine fever virus from pig farms in South Korea during outbreaks in 2019–2021. *Viruses*.

[29] Forth J. H., Calvelage S., Fischer M. (2023). African swine fever virus—variants on the rise. *Emerging Microbes & Infections*.

[30] Forth J. H., Tignon Mène, Cay A. B. (2019). Comparative analysis of whole-genome sequence of African swine fever virus Belgium 2018/1. *Emerging Infectious Diseases*.

[31] Lu Y., Deng X., Chen J., Wang J., Chen Q., Niu B. (2019). Risk analysis of African swine fever in Poland based on spatio-temporal pattern and Latin hypercube sampling, 2014–2017. *BMC Veterinary Research*.

[32] Sauter-Louis C., Forth J. H., Probst C. (2021). Joining the club: first detection of African swine fever in wild boar in Germany. *Transboundary and Emerging Diseases*.

[33] Fernández-Pinero J., Gallardo C., Elizalde M. (2013). Molecular diagnosis of African swine fever by a new real-time PCR using universal probe library. *Transboundary and Emerging Diseases*.

[34] King D. P., Reid S. M., Hutchings G. H. (2003). Development of a TaqMan® PCR assay with internal amplification control for the detection of African swine fever virus. *Journal of Virological Methods*.

[35] Suchard M. A., Lemey P., Baele G., Ayres D. L., Drummond A. J., Rambaut A. (2018). Bayesian phylogenetic and phylodynamic data integration using BEAST 1.10. *Virus Evolution*.

[36] Rambaut A., Drummond A. J., Xie D., Baele G., Suchard M. A., Susko E. (2018). Posterior summarization in Bayesian phylogenetics using tracer 1.7. *Systematic Biology*.

[37] Kim G., Park J.-E., Kim S.-J. (2022). Complete genome analysis of the African swine fever virus isolated from a wild boar responsible for the first viral outbreak in Korea, 2019. *Frontiers in Veterinary Science*.

[38] Pérez-Losada M., Arenas M., Galán J. C., Palero F., González-Candelas F. (2015). Recombination in viruses: mechanisms, methods of study, and evolutionary consequences. *Infection, Genetics and Evolution*.

[39] Zhu Z., Xiao C.-T., Fan Y. (2019). Homologous recombination shapes the genetic diversity of African swine fever viruses. *Veterinary Microbiology*.

[40] Li X., Xiao K., Zhang Z. (2020). The recombination hot spots and genetic diversity of the genomes of African swine fever viruses. *Journal of Infection*.

[41] Zhao D., Sun E., Huang L. (2023). Highly lethal genotype I and II recombinant African swine fever viruses detected in pigs. *Nature Communications*.

[42] Dixon L. K., Chapman D. A. G., Netherton C. L., Upton C. (2013). African swine fever virus replication and genomics. *Virus Research*.

