# Supplementary material for: Genomic Epidemiology of African Swine Fever Virus Identified in Domestic Pig Farms in South Korea during 2019–2021
Source: Transbound Emerg Dis. 2024 Jan 16;2024:9077791. doi: 10.1155/2024/9077791 (PMC12016747; doi:10.1155/2024/9077791)
Supplement: Supplementary Material — Table S1: primers for Sanger sequencing of partial MGF-505-9R. Table S2: list of reference African swine fever virus sequences used in phylogenetic analysis. Table S3: summary of genetic variations detected between 21 Korean African swine fever viruses sequences and the reference strain, Georgia 2007/1 (NC_044959). [file 9077791.f1.docx]

Supporting materials

**Genomic surveillance of African swine fever viruses detected in domestic pig farms: Insights from whole genome sequences in South Korea during 2019-2021**

Oh-Kyu Kwon^1†^, Da-Won Kim^2†^, Jin-Hwa Heo^1^, Ji-Yun Kim^2^, Jin-Ju Nah^1^, Ji-Da Choi^1^, Dong-Wook Lee^2^, Ki-Hyun Cho^1^, Seong-Geun Hong^1^, Yeon-Hee Kim^1^, Hae-Eun Kang^1^, Jung-Hoon Kwon^2*^, and Yeun-Kyung Shin^1*^

^1^Foreign Animal Disease Division, Animal and Plant Quarantine Agency, Gimcheon, Gyeongsangbuk-do, Republic of Korea

^2^College of Veterinary Medicine, Kyungpook National University, Daegu, Republic of Korea

^†^ Two authors contributed equally.

S1 Table. Primers for sanger sequencing of partial MGF-505-9R

| **Target** | **Primer** | **Sequence** | **Length** |
| --- | --- | --- | --- |
| MGF 505-9R | ASFV_43697_F | TACTGGGAAGGCCATGGTTCAGT | 400bp |
|  | ASFV_44122_R | TCGGCATGCCATCTCATACAGAA |  |

S2 Table. List of reference African swine fever virus sequences used in phylogenetic analysis

| **Strain name** | **Length** | **Country** | **Collection_Date (YYYY-MM-DD)** | **Genbank Accession Number** |
| --- | --- | --- | --- | --- |
| ASFV Georgia 2007/1 | 190,584 | Georgia | 2007 | NC_044959 |
| 20355/RM/2022_Italy | 190,590 | Italy | 2022 | OP605386 |
| Korea/HC224/2020 | 188,645 | South Korea | 2020 | OP628183 |
| SY-2 | 189,404 | China | 2021-10-01 | OP612151 |
| 2021ASP01919 | 190,595 | Germany | 2021-04-19 | OX376251 |
| 2021ASP00902 | 190,595 | Germany | 2021-02-18 | OX376255 |
| 2021ASP02148 | 190,595 | Germany | 2021-04-29 | OX376257 |
| 2021ASP02665 | 190,611 | Germany | 2021-05-11 | OX376259 |
| 2020ASP02103 | 190,595 | Germany | 2020-10-07 | OX376261 |
| 2021ASP00921 | 190,597 | Germany | 2021-02-22 | OX376262 |
| 2021ASP03740 | 190,592 | Germany | 2021-07-29 | OX376272 |
| SY-1 | 189,404 | China | 2020-06-01 | OM161110 |
| ASFV/Kaliningrad_17/WB-13869 | 189,129 | Russia | 2017-11-07 | OM799941 |
| ASFV/Kaliningrad_18/WB-12523 | 189,111 | Russia | 2018-08-07 | OM966714 |
| ASFV/Kaliningrad_18/WB-12524 | 189,133 | Russia | 2018-07-30 | OM966715 |
| ASFV/Kaliningrad_18/WB-9763 | 189,125 | Russia | 2018-07-07 | OM966717 |
| ASFV/Kaliningrad_19/WB-10168 | 189,131 | Russia | 2019-05-13 | OM966719 |
| ASFV/Kaliningrad_18/WB-12516 | 189,143 | Russia | 2018-08-07 | OM966720 |
| ASFV/Kaliningrad_18/WB-9734 | 189,127 | Russia | 2018-06-25 | OM966721 |
| GZ201801_2 | 189,401 | China | 2018-12-22 | ON263123 |
| African swine fever virus serotype 8 genotype 2 | 189,487 | Viet Nam | 2021-10-01 | ON402789 |
| A4 | 192,377 | Philippines | 2021-08-01 | ON963982 |
| Yangzhou | 187,951 | China | 2021-11-01 | ON456300 |
| Korea/YC1/2019 | 188,950 | South Korea | 2019 | ON075797 |
| ASFV/Zabaykali/WB-5314/2020 | 189,248 | Russia | 2020-08-04 | MZ325862 |
| 2802/AL/2022 Italy | 190,596 | Italy | 2022 | ON108571 |
| ABTCVSCK_ASF001 | 190,598 | India | 2020 | OM481275 |
| ABTCVSCK_ASF007 | 190,595 | India | 2021 | OM481276 |
| IND/AS/SD-02/2020 | 190,517 | India | 2020-04-01 | OL692743 |
| IND/AR/SD-61/2020 | 190,572 | India | 2020-04-01 | OL692744 |
| ASFV-wbShX01 | 189,401 | China | 2019-11-01 | MW033528 |
| CADC_HN09 | 190,257 | China | 2019 | MZ614662 |
| ASFV/Amur 19/WB-6905 | 189,248 | Russia | 2019-08-29 | MW306190 |
| ASFV/Primorsky 19/WB-6723 | 189,256 | Russia | 2019-08-28 | MW306191 |
| ASFV/Timor-Leste/2019/1 | 192,237 | Timor-Leste | 2019 | MW396979 |
| ASFV Germany 2020/1 | 190,592 | Germany | 2020 | LR899193 |
| ASFV/Kabardino-Balkaria 19/WB-964 | 189,252 | Russia | 2019-03-26 | MT459800 |
| Pol17_55892_C754 | 189,414 | Poland | 2019 | MT847620 |
| Pol18_28298_O111 | 189,409 | Poland | 2019 | MT847621 |
| Pol17_31177_O81 | 189,422 | Poland | 2019 | MT847622 |
| GZ201801 | 189,393 | China | 2018-12-22 | MT496893 |
| CN/2019/InnerMongolia-AES01 | 189,403 | China | 2019-02-19 | MK940252 |
| ASFV Wuhan 2019-1 | 190,576 | China | 2019-08-19 | MN393476 |
| ASFV Wuhan 2019-2 | 190,576 | China | 2019-08-19 | MN393477 |
| ASFV_HU_2018 | 190,601 | Hungary | 2018-04-24 | MN715134 |
| ASFV/pig/China/CAS19-01/2019 | 189,405 | China | 2019-01-02 | MN172368 |
| ASFV/LT14/1490 | 189,399 | Lithuania | 2014-01-01 | MK628478 |
| ASFV Moldova 2017/1 | 190,598 | Moldova | 2017 | LR722599 |
| ASFV CzechRepublic 2017/1 | 190,594 | Czech Republic | 2017 | LR722600 |
| Belgium/Etalle/wb/2018 | 190,202 | Belgium | 2018-09-10 | MK543947 |
| ASFV-wbBS01 | 189,394 | China | 2018-11-01 | MK645909 |
| ASFV Belgium 2018/1 | 190,599 | Belgium | 2018 | LR536725 |
| Pig/HLJ/2018 | 189,404 | China | 2018-09-05 | MK333180 |
| DB/LN/2018 | 189,404 | China | 2018-09-01 | MK333181 |
| China/2018/AnhuiXCGQ | 189,393 | China | 2018-09-02 | MK128995 |
| Georgia 2008/1 | 189,465 | Georgia | 2008 | MH910495 |
| Pol16_20186_o7 | 189,401 | Poland | 2017-12-01 | MG939583 |
| Pol17_04461_C210 | 189,401 | Poland | 2017-12-01 | MG939588 |
| Pol17_05838_C220 | 189,393 | Poland | 2017-12-01 | MG939589 |
| ASFV/POL/2015/Podlaskie | 189,394 | Poland | 2015 | MH681419 |
| Estonia 2014 | 182,446 | Estonia | 2014 | LS478113 |
| Kashino 04/13 | 189,387 | Russia | 2013-03-01 | KJ747406 |
| ASFV Georgia 2007/1 | 190,584 | Georgia | 2007 | FR682468 |
| Arm/07/CBM/c2 | 190,145 | Armenia | 2007 | LR812933 |

S2 Table. Summary of genetic variations detected between 21 Korean African swine fever viruses sequences and the reference strain, Georgia 2007/1 (NC_044959)

| **Gene** | **Variant (SNP, deletion, insertion)** | **Amino acid change** |
| --- | --- | --- |
| MGF 360-1 La | A2329G | L106P |
| MGF 360-1 Lb, MGF 360-2L IGR^A^ | 2962 T deletion ( T 11>9) | non-coding |
| MGF 360-1 Lb, MGF 360-2L IGR | 2962-2963 T deletion ( T 11>10) | non-coding |
| MGF 360-2L, KP177R IGR | 4077/8 T insertion (T 9>10) | non-coding |
| MGF 360-3L, MGF 110-1L IGR | 6783 T deletion (T 9>8) | non-coding |
| MGF 110-1L | C7059T | W197* (215>197)^B^ |
| MGF 110-5L-6L, MGF 110-7L IGR | G10217A | non-coding |
| MGF 110-7L | G10388A | P114S |
| 285L | G11277A | A17V |
| ASFV G ACD 00190 | 12578 A deletion (A 10>9) | frame shift and protein truncation (42>25) |
| MGF 360-4L | C16649G | V243L |
| MGF 360-4L, ASFV G ACD 00300 IGR | 17846/7 G insertion (G 9>10) | non-coding |
| ASFV G ACD 00320, 00330 IGR | 19799 G deletion (G 8>7) | non-coding |
| X69R | 20405/6 CTA insertion | Y insertion (70>71) |
| MGF 300-1L MGF 300-2L IGR | 21832 A deletion | non-coding |
| MGF 300-4L | G23149A | A251V |
| MGF 360-10L | T26425C | N329S |
| MGF 360-10L | C27183G | Syn^C^(L) |
| MGF 360-12L | G30538A | Syn(F) |
| MGF 360-12L | G30606A | L268F |
| MGF 360-14L | 33042/3 C insertion (C5>6) | frame shift and protein truncation (358>288) |
| MGF 505-3R | 36146 C deletion | frame shift and protein truncation (281>134) |
| MGF 505-9R | 43883 ~ 43934 multiple mutation | R92D, Q93R, Syn(D/95), Syn(L/96), Syn(I/97), Q98H, Syn(Y/101), N102D |
| MGF 505-9R | A44576G | K323E |
| K145R, K421R IGR | 66175/6 T insertion (T9>10) | non-coding |
| EP1242L | G70064C | Syn(R) |
| B602L, B385R IGR | 103315/6 G insertion | non-coding |
| CP530R | A127208G | M271V |
| NP419L | T134514C | N414S |
| NP868R | C137334T | L506F |
| P1192R | C150928A | Syn(I) |
| I267L | T170862A | I195F |
| I73R, I329L IGR | GAATATATAG insertion | non-coding |
| MGF 505-11L | G179863A | Syn(S) |
| ^A^ Intergenic region, ^B^(X>Y) X, the number of amino acid before frame shift; Y, the number of amino acid after frame shift and protein truncation, ^C^ Synonymous mutation, *stop codon, Abbreviations: A, Alanine; N, Asparagine; I, Isoleucine; L, leucine; K, Lysine; M, Methionine; F, Phenylalanine; P, Proline; S, Serine; W, Tryptophan; V, Valine; R, Arginine | | |
